# Supplementary figures and images for: Reduction of coastal lighting decreases seabird strandings
Source: PLoS One. 2024 Jun 5;19(6):e0295098. doi: 10.1371/journal.pone.0295098 (PMC11152301; doi:10.1371/journal.pone.0295098)

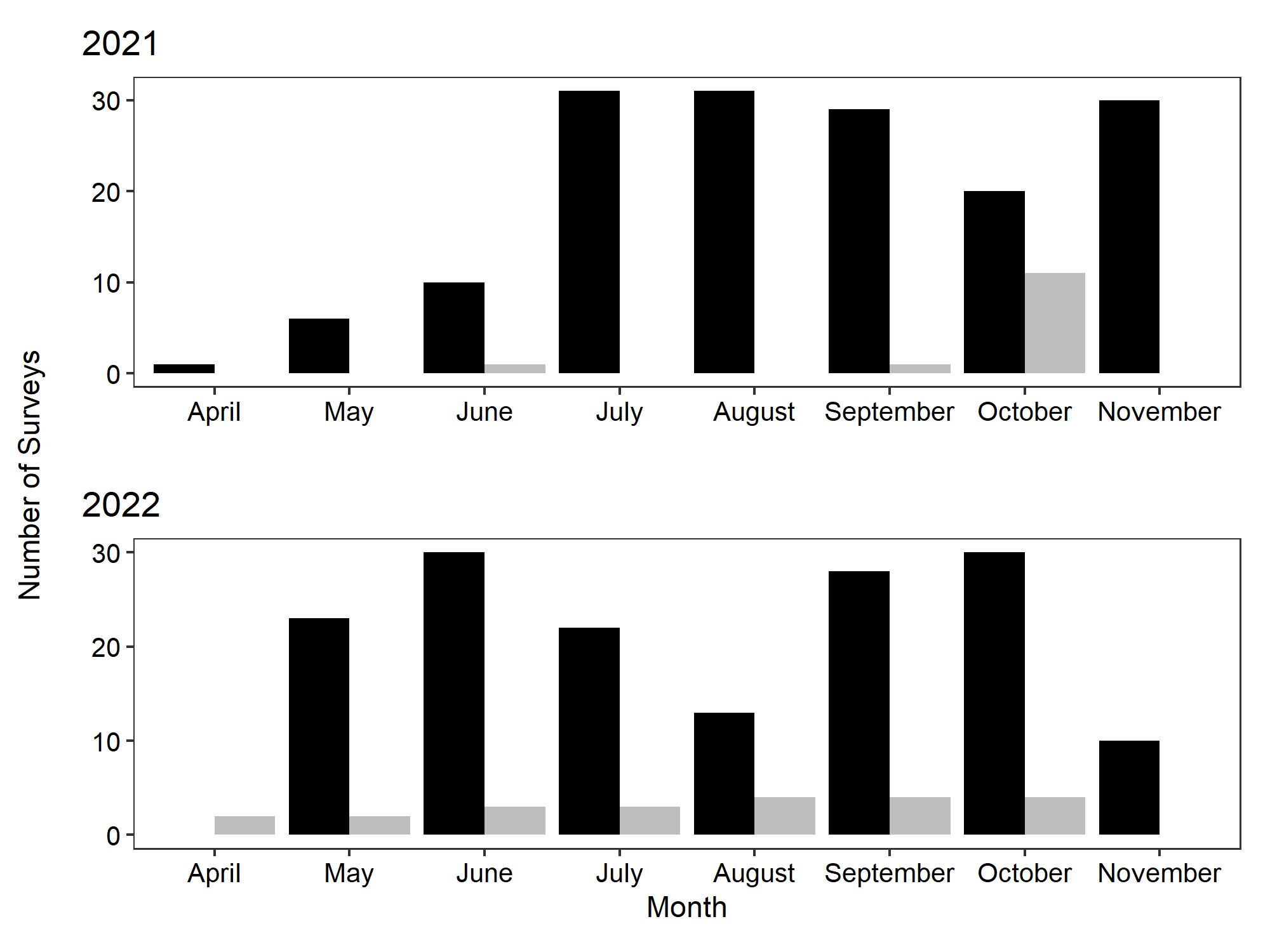

Supplement: S1 Fig — (TIF) [file pone.0295098.s001.tif]

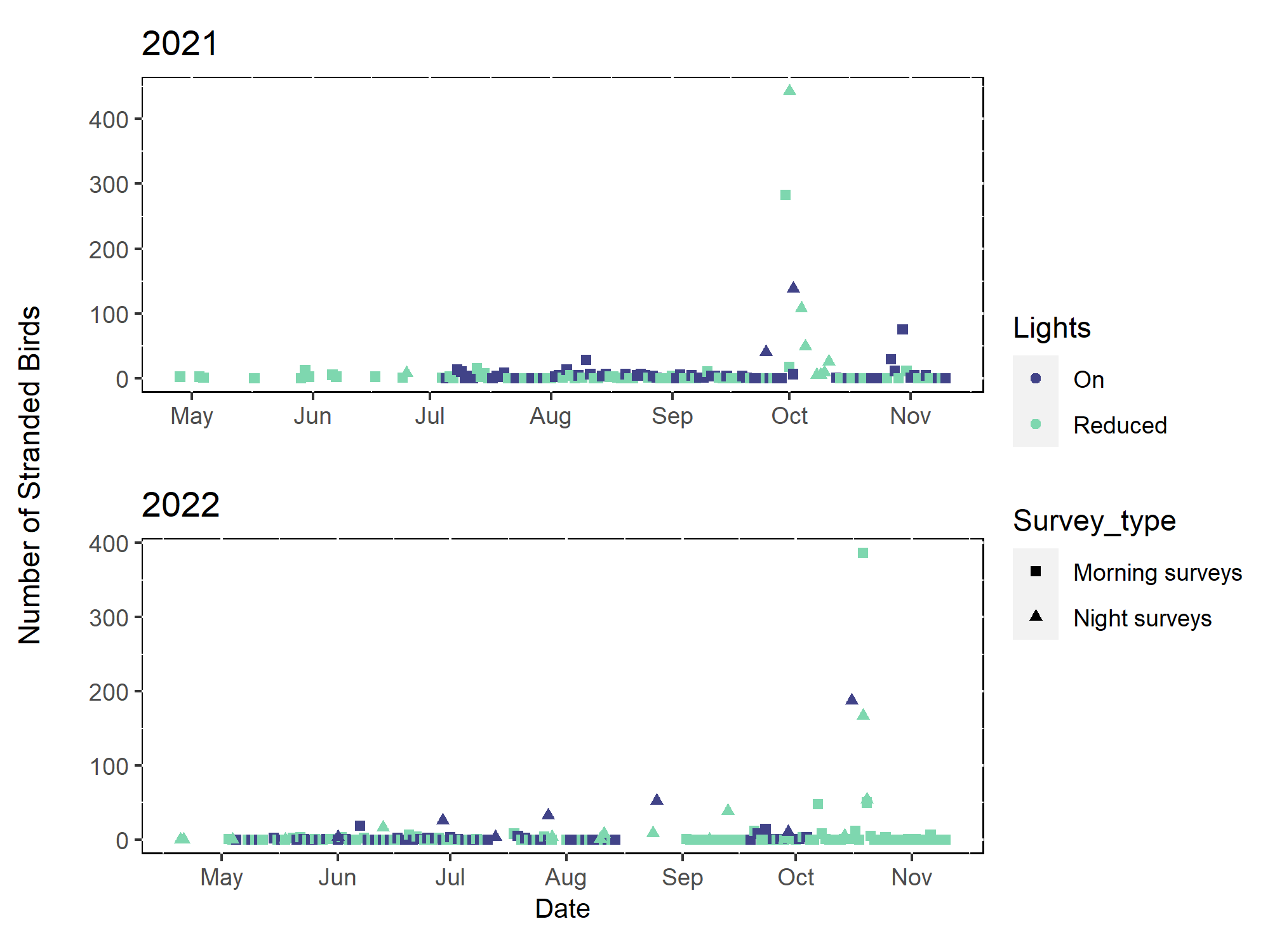

Supplement: S2 Fig — (TIF) [file pone.0295098.s002.tif]

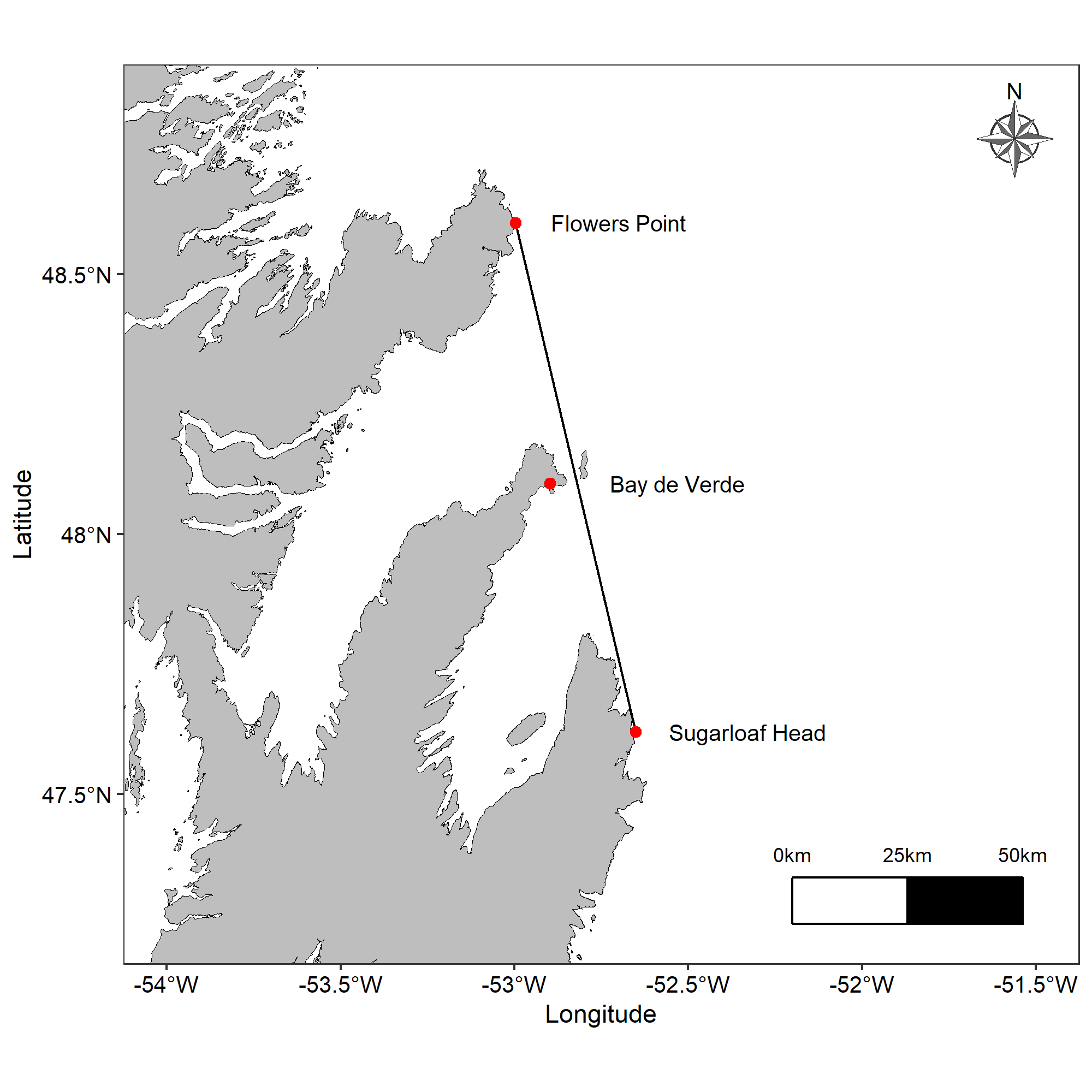

Supplement: S3 Fig — The bearing angle between Flowers Point (166°) and Sugarloaf Head (346°) was calculated relative to North (0°) using the black tangent line. This angle was used to classify wind direction as either onshore (less than 166° and greater than 346°) or offshore (greater than 166° and less than 346°). (TIF) [file pone.0295098.s003.tif]
