# Supplementary material for: Reduction of coastal lighting decreases seabird strandings
Source: PLoS One. 2024 Jun 5;19(6):e0295098. doi: 10.1371/journal.pone.0295098 (PMC11152301; doi:10.1371/journal.pone.0295098)
Supplement: S1 Text — (DOCX) [file pone.0295098.s004.docx]

On the nights of September 13, 28, 29, and October 14 2022, identifiable Leach’s Storm-Petrel body parts (i.e. wings, legs, back, breast, etc.) were counted and removed from the perimeter of the plant to approximate a measure of mortality that occurred between collection dates when researchers were not present. Since body part counts were only performed opportunistically in September and October and because this period corresponds with previously reported peak stranding periods [[3,8,10]](https://www.zotero.org/google-docs/?xhLiid), these results cannot be generalized to approximate mortality when researchers were not present throughout the entire breeding season. Consequently, we only present results for the number of mortalities that likely occurred from September 13 to October 14 2022.
