# Supplementary material for: Reduction of coastal lighting decreases seabird strandings
Source: PLoS One. 2024 Jun 5;19(6):e0295098. doi: 10.1371/journal.pone.0295098 (PMC11152301; doi:10.1371/journal.pone.0295098)
Supplement: S2 Text — (DOCX) [file pone.0295098.s005.docx]

We devised a formula for calculating mortality per night at the plant. When body parts (Bp) were identifiable as belonging to a single individual (e.g., a pair of wings, a pair of legs, bodies, and/or heads) they were counted as one bird. Singular wings (W) each accounted for half of one bird, as separate singular wings could be from one individual or 2 separate individuals. Days was defined as the number of days between each collection period when researchers were not present at the plant. The formula for mortality per night (M_n_) is then: M_n_ = (Bp + (W/2)) / days

To calculate an average of the number of birds that perished per night from September 13 to October 14, 2022, we summed the mortality per night for each of the three collection periods (September 13 to September 28 – 79 full body parts and 70 wings, September 28 to September 29 – 15 full body parts and 15 wings, September 29 to October 14 – 24 full body parts and 20 wings) and divided it by the number of collection periods. To calculate total mortality from September 13 to October 14, 2022, we multiplied average mortality per night by the number of nights in the collection period. Body parts from the night of September 13 were excluded in these analyses.
