# Supplementary material for: Reduction of coastal lighting decreases seabird strandings
Source: PLoS One. 2024 Jun 5;19(6):e0295098. doi: 10.1371/journal.pone.0295098 (PMC11152301; doi:10.1371/journal.pone.0295098)
Supplement: S3 Text — (DOCX) [file pone.0295098.s006.docx]

Based on four nights of counting storm-petrel body parts, we estimated that from September 13 to October 14, 2022, 300 birds died when researchers were not present.
