# Supplementary material for: Reduction of coastal lighting decreases seabird strandings
Source: PLoS One. 2024 Jun 5;19(6):e0295098. doi: 10.1371/journal.pone.0295098 (PMC11152301; doi:10.1371/journal.pone.0295098)
Supplement: Attachment — Submitted filename: review-of-artificial-light-at-night-and-seabird-strandings.docx [file pone.0295098.s007.docx]

Burt et al. (2023) paper review

**Summary of the Research**

In this study, the authors conducted an experiment whereby the exterior lights at a seafood processing plant in Newfoundland were manipulated in two different treatments (“on” and “off”) to test the hypothesis that Leach’s Storm-Petrels become stranded there each year due to positive phototaxis. They used two survey methods (nocturnal, during stranding; and diurnal, following stranding) to quantify the number of Leach’s Storm-Petrel strandings that occurred over two years and modeled the effects of light treatment as well as moon phase and weather on stranding numbers. They also took steps to quantify mortality of stranded birds and estimate the proportion of stranded birds belonging to each age class (juvenile or adult). The authors found that more birds became stranded in the lights-on treatment, when moon illumination was low, in the first year of the study, and later in the year. Most stranded birds were juveniles, mortality of stranded birds was around 14%, and the peak stranding period occurred from the end of September to the end of October. Given these results, the authors recommend the extinguishing of artificial lights at night wherever possible in locations near Leach’s Storm-Petrel breeding colonies, and increased public education and involvement in the effort to reduce stranding-related mortality.

**Major comments**

I greatly enjoyed reading the manuscript and feel that it is a worthy contribution to the literature. Considering the number of seabird species in the world that are affected by stranding and its resulting mortality, it is heartening to learn through the efforts of the authors that certain very simple actions (that is, the turning off of lights) can significantly reduce stranding numbers. I have no critical issues with the manuscript, although I have outlined below some general changes that I would recommend for the text at large, followed by minor comments. Most importantly to me, I would like to see the final light treatment schedule and how it aligns with the schedule of both survey types used.

Please note that there does not appear to be any public repository on Github under the username of “tori-burt”.

Light treatments are called “on” and “off” throughout the manuscript; however, because not all the lights were turned off at the seafood processing plant in the “off” treatment, I would suggest following the example of Miles et al. (2010) in calling the light treatments “on” and “reduced”. Although it seems like a minor change, someone skimming the paper might otherwise interpret the overall results as “strandings were reduced – but not eliminated – when lights were [completely] turned ‘off’”. Whereas, it’s more accurate to say that strandings were reduced when artificial lighting was reduced, which further suggests that perhaps strandings could have been eliminated if artificial lighting had been eliminated (so as to actually emphasize the importance of the manuscript’s results, rather than diminish it).

Additionally, it would be very helpful to me as a reviewer in assessing the study’s methodology at large and replicability in particular if a detailed (i.e., nightly) breakdown was provided of which treatment was applied (lights “on” or “reduced”) each night. E.g., this could be accomplished with a two-panel figure (one panel for each year), perhaps in the Supporting Information (including, ideally, the nights that nocturnal surveys were conducted). The authors mention there were some extended periods of the “reduced” light treatment (e.g., when the plant was shut down) which understandably could not have been avoided, but in its current form the manuscript does not give me a clear idea of the final lighting treatment schedule or of just how much of an effect these longer “reduced” lighting periods might have had on the results.

**Minor comments**

I have provided some more specific comments on a line-by-line basis below that I hope the authors will find helpful.

L30. This seems to imply that “positive phototaxis” is defined as or synonymous with “movement towards artificial light at night” when it could refer to movement toward any light at any time. I suggest minor rephrasing

L47-49. This sentence is good but there is a lot to unpack and it could benefit from a (brief) follow-up sentence or two explaining the perils related to stranding / rates of mortality following stranding. But this is very minor.

L61. I might suggest also citing Imber (1975; Notornis) here (in addition to citation #15)

L74-76. I just wanted to say that these are both extremely good points, and that is why it’s so great that the authors have done this current study!

L77. Spelling: “phototactic” vs. “phototaxic”

L79-81. I might suggest a slight reformatting / rewording of this sentence to ensure it’s as clear as possible.

L106-107. I also mention this with regard to the figures, but I think it is worthwhile outlining the entire (or at least all of the major parts of) the lighting scheme of the whole building; not just the lights that were manipulated for the purposes of the experiment. I say this in part because from what I understand, petrels were picked up all around the building, not just on the NW side where the lights were manipulated (unless I am incorrect in my understanding of any of this). So the counts of birds stranded include presumably those affected (i.e., attracted) by the lights on the back of the building, and by the lights on the front of the building that remained “on” even in the “off” (reduced) treatment. So I think these un-manipulated lights are still relevant.

L123-24. Would it be possible to make the entire schedule (complete with each day’s actual rather than planned lighting treatment) available as Supporting Information, for the purposes of reviewing how balanced the schedule was?

L124. For how long in total / how many 28-day periods in total (over the course of each year)?

L125-26. But did they, in fact, follow the schedule? Or did some nights deviate from the plan?

L127-28. Similar to previous comments, this is where a schedule outlining the actual lighting treatment applied each night would be helpful as Supplementary Information. Otherwise it’s difficult to evaluate how well the lighting schedule actually allows the authors to test the hypotheses and whether there are any potential biases in the data as a result of lighting schedules (even if deviations from the schedule could not be helped)

L128. How many lights are also on the back of the building? And how many lights were left on at the front of the building even during “dark” treatment nights? (As an aside, these various light locations could be shown in Figure 2 with a different symbol to represent the fact that they were always on)

L129. Is it known what “briefly” constitutes here, on average or at maximum, roughly? Just out of curiosity.

L147. “the following evening” – to clarify: this is two evenings after stranding / capture?

L149. Weather conditions on the morning of collection, or on the [previous] night of stranding? And including which variables exactly, and how were they measured?

L149-50. Were birds collected from vessels? Or is this noted to determine when the lights were turned on? It might be useful to explain the significance of collecting vessel information at some point in the Introduction.

L153-54. This point makes it especially of interest for me / readers to know the final lighting treatment schedule.

L157. What is the total estimated capacity of all the bird shelters combined? (this becomes useful information later, when considering predation that occurs before morning surveys / bird collection)

L159-60. At the end of this line, it’s stated that there were 22 night surveys conducted in 2022; how many were conducted in 2021?

Also, based on these lines it appears the timing of nocturnal surveys differed by year where in 2021 they were opportunistic and in 2022 they were approximately bi-weekly; but in the rest of the manuscript, these nocturnal surveys (from both years) appear to be collectively referred to as bi-weekly. I suggest omitting the term “bi-weekly” when collectively referring to the nocturnal surveys since it does not accurately describe the timing of nocturnal surveys for both years and since it could mean one of either “twice per week” or “twice per month” without the definition being provided.

L157-84. It would be pertinent to state somewhere in this section how many night surveys were conducted during lights “on” versus “reduced”, in both years. Because the method for deciding when to do nocturnal surveys differed between years, I would be concerned about one year having the surveys coincidentally falling on more lights “on” nights than “reduced” nights, and possibly affecting the results.

L164. What was it about the condition of the feathers that indicated they were juveniles?

L171-72. Please clarify if possible, as I’m not sure I understand the meaning here, in the context of the rest of the sentence.

L177-78. Same as in the daily survey methods section, I would like to know the relevance of including the vessel data.

L179. Repeats from lines 159-60; could be combined / condensed

L182. Is it not known exactly how long the lights were off for this time period? If not known, how were daily survey stranding numbers matched to the treatment used?

L189. Please clarify if this includes birds found both alive and dead.

L191-92. Is this an established method used in (an)other study/ies for which a citation can be provided? Or how was this method decided upon, to determine the peak stranding period?

L193-211. Please clarify that in this analysis, only daily / morning survey data were used.

L201-02. The data used were from which location (St. John’s?) and at which time point? Also, this sentence says “weather information” is from Custom Weather Inc, but once wind speed and direction are removed (which were apparently obtained from the government of Canada), the only weather variable left in the above list of variables is fog; was this the only variable gleaned from Custom Weather Inc? If so, the fog data originate from what location? I am partly curious because fog can be extremely localized and I would be very cautious about taking fog data from St. John’s to apply to Bay de Verde.

L203-04. Wind data are for St. John’s, or elsewhere? And wind data were used from which time point(s) – a certain time of the night during which strandings occurred (i.e., the night before bird collection)?

L201-06. So, just to be clear, none of the weather data collected on the mornings of bird collection were used in the analyses (see line 149)? If this is the case, I might suggest omitting mention of collecting these data at all (and omit mention of any other data collected that were not used, if applicable).

L209-10. Were these events (>100 birds) considered statistical outliers? Or how was “outlier” defined? How many of these events were there over the course of the study, just to give some context?

L212. Please clarify if this analysis includes birds found both alive and dead.

L214. It is stated in line 178 that fog data were collected during these surveys; please double-check.

L215. What is considered “supplementary data”, and how were they used to estimate cloud conditions? Or were the St. John’s cloud data simply used as-is?

L218. See comment re: “outliers”, lines 209-10.

Results. The p-value column(s) in the two tables contain a mixture of exact p-values and significance levels, and the way they are reported in tables does not always match the way they are reported in-text. Please double-check consistency and ideally state somewhere which significance level you used as a cutoff.

L223. I believe the chi-square value of 6.73 might actually round to 6.74 based on Table 1. Also I suggest references to the appropriate tables be made in the brackets with test statistics, where applicable.

L224. Is it a 95% confidence interval that’s being presented? If so, I would suggest adding “95%” before the “CI”, in the brackets (and throughout, where applicable; e.g., L246).

L228-29. Since this sentence is about “birds stranded per night” being higher in 2021 than in 2022, might it be more appropriate, instead of reporting total strandings per year, to report the average number of birds per night?

L229-30. Fog – from Table 1, it does appear that fog is significantly associated with the number of stranded birds, at least to p < 0.001, unless I am misunderstanding the results. Please double-check.

L231. I would suggest perhaps including the test statistic(s) and p-value here as well as in the table. (See also L251)

Table 1. If applicable, I suggest clarifying in the caption that this table presents results from the daily / morning surveys only

L245. How are the “later months of the breeding season” defined for the analysis? I suggest detailing this in Methods > Statistical Analysis, unless it actually refers to “day of year”, in which case I would suggest changing the wording here to specifically reflect that.

Table 2. Similar to Table 1, I suggest clarifying in the caption that this table presents results from the nocturnal surveys only

L262-63. Similar to comment on L245, I might suggest using slightly more precise wording so as to avoid confusion. I assume “seasonal effect” refers to the effect of “day of year” on stranding numbers, since there was no variable based on “season” used in the models.

L266-67. “Body part estimations” have not been introduced or explained in the main text of the manuscript thus far. If the authors want to keep it in the main text, I would suggest bringing the methodology over from the Supporting Information to the main text. However, I would actually instead suggest omitting it from the main text. In this case, including body part estimations (which were only conducted four times during the whole study and half of those times during the peak stranding period) in calculating the percentage of birds stranded during the “peak stranding period” seems to me like it would only introduce unnecessary noise into the calculation.

L271. Does this “error” refer specifically to Standard Error?

L272. “outliers” – see comment L209-10.

L276. Is “body part count” the same as body part estimation? How defined? See comment on L266-67.

L278-79. Similar to comment on L266-67, I feel that including body part counts in this calculation of % alive only adds unnecessary noise, since the body part counts were not conducted during every survey.

L279-80. When I add 1950 (birds stranded when lights on) and 1929 (birds stranded when lights reduced), I get 3879 birds total. Even if I add to this the 300 birds estimated from the body part counts, I only come to a total of 4179 birds. I’m not sure if I am doing the math incorrectly, but this does not equal the total given in L277 of 4225 birds; perhaps a bit of clarification would be helpful.

L282-84; Table 3. I would suggest specifying here exactly how many birds were “examined” in total (including, presumably, not only adults and juveniles but also unknown-age birds). As written, it appears that a total of 1859 birds were aged (1609 juveniles + 250 adults), and all of them were assignable to either juvenile or adult (because 86.6% + 13.4% = 100%). But in that case it becomes unclear where the number 731 comes from for “unidentified” (unknown age) birds in Table 3. I also suggest changing “examine” (L283) to “assign age to” (or something similar), for clarity.

One other thought / question I have is, could the bias in 2021 toward conducting nocturnal surveys during the peak stranding season (when it is known they are mostly juveniles) cause a bias toward proportionally more juveniles than if nocturnal surveys had been conducted evenly throughout both 2022 and 2021?

L284. What percentage of adults stranded from June to August?

L288-89. How are these time periods defined exactly (“fall” and “breeding season”) for the purposes of these results? Does this mean the nocturnal surveys from June 2021 are omitted (and if so, why)? If “fall” and “breeding season” are not strictly relevant, I would suggest omitting and just saying 2021 and 2022.

Tables 3 and 4; also throughout. For clarity, I suggest changing “unidentified” to “unknown-age”.

L300-01. In referring to such a small number of nights of mass strandings (8 total), instead of using % (e.g., 62.5%, 75%) I would suggest using raw / whole numbers.

L302-03. Is it meant here that 6/8 mass stranding events occurred on nights of nocturnal surveys, and 2/8 occurred based on the more limited evidence of morning surveys? I’m curious what the authors think about the fact that morning surveys are a different methodology that misses a lot of the birds that come in, therefore probably “missing” some nights that would otherwise be considered “mass stranding events” (>100 birds).

L316-17. It is unclear to me what the main take-home message is of this sentence without knowing how this statistic relates or compares to the other years of that study (i.e., without additional context). Were 1982 and 1983 exceptional somehow in the proportion of shearwaters found in brightly illuminated locations? Or was the proportion high in all years? Are the years relevant, or could an overall proportion be provided for the entire study?

L317-19. That any number of birds was grounded anywhere does not suggest positive phototaxis in itself; I suggest the authors add some sort of direct context related to light that would support the suggestion of positive phototaxis more directly.

L319-20. I suggest omitting or altering the first clause of this sentence (before the semicolon) because the meaning is somewhat unclear. I initially interpreted it as either the population increasing as the result of reduced ALAN, or that they responded with attraction (“positive” phototactic behaviour) toward reduced ALAN; only after reading the rest of the sentence do I realize that neither of those meanings was intended by the authors.

L322-23. Please consider adding a couple words to specify that this sentence refers to the current study.

L324. I understand from the Methods and Results that cloud cover data were indeed obtained and used in analyses. Please double-check and/or clarify this sentence based on that.

L336-39. I think this sentence could benefit from rephrasing for better flow and clarity.

L341-44. This is very interesting! I would genuinely be curious as to what the authors suggest as a good next step in the research on this topic. Do the authors have a hypothesis underlying this point about the relationship between cloud cover / fog and mass strandings?

L345-70. The train of thought in this (quite large) paragraph seems to me to be somewhat murky in the sense that I’m not sure what the main take-home message(s) is/are, and the main topic seems to shift throughout. I suggest breaking into 2+ separate paragraphs, each with its own take-home message; e.g., one on mortality, survival, predation levels, and how nocturnal surveys capture a different picture of stranding numbers than morning surveys; and another on fledglings versus adults being differentially affected by light and why, taking into particular consideration the fact that mass stranding events occur just as often (or more often) in reduced lighting conditions.

L345. I’m not sure that including “breeding season” here is necessary, as it might just add confusion for the reader. The entire time storm-petrels are present in the area is the breeding season.

L347-50. I suggest briefly describing the implication of this finding.

L345-54. These lines could probably be condensed and made more concise, and some additional discussion of the body part counts could be both interesting and relevant here (if desired).

L356. “season” – see comment on L245.

L356-57. Please specify whether this is generally true across latitudes / breeding locations, or if it’s specific to Newfoundland.

L360. Please clarify if “these stranding events” refers specifically to the 8 documented mass stranding events or to strandings in general.

L363-64. The current study already shows that a partial reduction in lighting is enough to reduce strandings; I wonder if “eliminate” would be more accurate than “reduce”.

L364-65. I’m not sure that I understand the rationale for the second hypothesis. It would be helpful if the authors could clarify how the results outlined in L360-61 support this hypothesis (as it is said in L362-63 that the results could be interpreted as support for it; but in L365-66 it is said that results concur with the first hypothesis).

L365-67. These lines appear to repeat what was said in L354-60. Please double-check.

L367-69. These lines appear to partially repeat what was said in L340-41; perhaps they can be combined / condensed.

L369-70. If I understand their intended meanings correctly, perhaps the forward slash and comma can be replaced with “÷” and “=”, respectively.

L373-74. This sentence could be taken to mean that the number of stranded breeding birds in general may have been underestimated, but I doubt that was the meaning intended by the authors since counts of found stranded birds are almost by definition underestimates. I suggest the wording be altered slightly to clarify the intended meaning to be about the proportion of stranded adult birds that were verified as breeders.

L375. It might be helpful here to tell the reader when hatching typically occurs in this species in Newfoundland

L371-83. It might be worth mentioning / reiterating in this paragraph the population declines at Baccalieu and/or the threatened status of the species, therefore reinforcing the importance of reducing adult mortality related to positive phototaxis

L385-89. Very cool study. It sounds to me like social media reports of stranded birds throughout the province could help researchers find those stranding-prone locations and times to target for conservation efforts. A more explicit connection between those two concepts could be made here.

L389-91. “Conservation efforts at the seafood processing plant… are effective elsewhere” implies that the efforts at the seafood processing plant are somehow affecting strandings and mortality at a different site. Please consider re-wording slightly.

L399. Please specify whether this finding was related to passerines only, and clarify if the attractive effects of blue and green light were not observed in clear conditions.

L397-402. Just a note here to clarify that different light sources are really just different spectra of light, the same way that blue, green, red, and white light (L398-400) are different spectra of light that can vary even within a “colour” of light; they are all just collections of wavelengths expressed at different intensities. So I’m not sure that distinguishing between “wavelength” and “light source” as two different variables that may affect attraction and stranding is fully accurate.

L405. “LESP” as an acronym has not been defined in the manuscript; please use full name or define the acronym.

L415-16. The Puffin and Petrel Patrol (PPP) itself is only a program, but it is run by a non-profit organization (CPAWS-NL).

Figure 1. Are both map panels oriented North-up? A North indicator would be useful.

Figure 2. See also comment on L106-07. A separate symbol to indicate the locations of un-manipulated lights (e.g., on the back of the building and along the wharf) would be helpful in allowing the reader to see by how much the lighting was reduced in the reduced (“off”) light treatment. Since bird shelters are located along the back of the building and birds were presumably also collected there, it seems pertinent to include this information (in part also for replicability).

Supporting Information

SI. L16. “to approximate a measure of mortality when researchers were not present” – please clarify that the intended meaning is to quantify mortality that occurred between each date of body part counts, and how accurate these counts might be given the possibility of body parts disappearing in the interim.

SI. L26-28. A brief description of what (generally speaking) is being calculated would be helpful to include before defining the “days” variable and the formula.

SI. L29. I suggest using a more precise term than “lost”.

SI. L23-32. Please define what a “collection period” is and, in L31, specify the number of collection periods used in the calculation. Also, were the body parts collected on September 13 excluded from the final calculations of estimated mortalities that occurred between September 13 and October 14? Some additional clarification of the calculations described in L29-32 would be helpful.

SI. L34-35. Would the authors please consider including the numbers of each type of body part found on each collection date, so that the reader can confirm the results make sense given the calculations described in SI L23-32.

Thank you for the pleasure of reading this great paper!

Taylor Brown
