# Supplementary material for: Reduction of coastal lighting decreases seabird strandings
Source: PLoS One. 2024 Jun 5;19(6):e0295098. doi: 10.1371/journal.pone.0295098 (PMC11152301; doi:10.1371/journal.pone.0295098)
Supplement: Attachment — Submitted filename: Response to Reviewers.docx [file pone.0295098.s008.docx]

Response to Reviewers - PONE-D-23-37663

**Dear Airam Rodríguez,**

**Thank you for all of your thoughtful and constructive comments. We believe this review has vastly improved the quality of the manuscript. To help with the evaluation of our manuscript, we have included our responses to comments in bold and line numbers at the beginning of each statement from the revised document.**

Airam Rodríguez

Major Comments:

1. You include the number of adults and fledglings as a response variable. However, response to light is different between adults and fledglings, but also depending on the type of bird activity (foraging, visiting the colony at flight, fledging or when they are on land). See different responses for adults, nestlings and fledglings in, for example, Syposz et al. 2021 Scientific Reports, Rodríguez et al 2022 Frontiers in Ecology and Evolution or Atchoi et al. 2023 Journal of Experimental Biology. So, my suggestion is to build separate models for adults and fledglings. Given that you might have some unaged birds during the fledging period, you could run two models: one conservative using just the fledglings, and another including fledglings + unaged birds. I bet the influence of explanatory variables will be the same. Alternatively, you might assume that all grounded birds during the fledging period are fledglings (95%!! According to your data in line 285).

**We have now modeled adults, and juveniles + unaged birds in two separate GLMs. The adult model showed an effect for both light and year. The juvenile model did not show a light effect but there was an association with wind direction (increased stranding when winds were blowing onshore). As well, there was a quadratic relationship between day of year and the number of stranded birds per night.**

2. You use GLMMs and include day of the year as a predictor, but you are not expecting a linear relationship (showed in figure 6). If you follow my previous suggestion, in the case of fledglings, you should use a quadratic term as done in Rodríguez et al 2014 Plos One. It is reasonable to think that fledging follows a quadratic term along the date. In the case of adults, you might include the simple term ‘day of the year’ as long as you are expecting that the number of grounded adults decline with the date.

**We have now included date as a quadratic term in the juveniles + unaged birds model and kept date as “Day of year” for the adult model.**

3. What is the random term in your GLMM? It is not stated in lines 193-211. Then in results, you have some figures from negative binomial generalized models, but they have not been explained in Methods. Is it a misunderstanding between GLMMs and GLMs?

**We have removed the word “mixed” from this sentence and throughout the manuscript to clarify that we are reporting GLMs.**

4. Mortality analyses. I am not sure why authors do not model mortality as a proportional response variable with a binomial model. You have the numbers of dead and alive birds, so authors can identify the variables affecting mortality rates. I would also separate adults from fledglings as variables influencing mortality rates might be different for both groups. See Rodríguez et al 2014 Plos One for a similar statistical procedure.

**We have modelled juveniles and adults separately as suggested. For the daily morning surveys, our numbers of dead birds are likely an underestimation, as cats and gulls (which are abundant at the fish plant) would have removed most of these carcasses during the night. Similarly, when we were conducting the night surveys, we were unable to recover all dead birds because they were removed by predators before we found them. Our data are therefore not an accurate reflection of the ratio of surviving to perishing stranded birds. We merely counted both to get the best estimate of the number of affected individuals we could. As for variables affecting mortality rates, we were unable to determine how most birds died when collecting data. Many birds that came near the fish plant did not collide with it but would instead become disoriented and trapped within the light catch basin. Birds often died by predation or were found days to weeks later (these individuals that we found were included in the dead bird counts). Therefore, we cannot separate birds that died by environmental factors from those that died of other means, and a model for this would not contain the most meaningful variables.**

5. Introduction is focussed on Leach’s storm petrel, which is great, but why do you think that Leach’s storm-petrel is going to be so different to other seabird species? Given that seabird attraction to light is a common phenomenon to underground nesting seabirds (and with multiple similarities among the species involved), I would try to widen the scope to seabirds in general (you already did it in the title!). You might include all the specific information of the Leach’s storm petrel (e.g. lines 51-58 or 61-62), in a section entitled Model species or Study species (or something like that). Then, in lines 59-83, every time you talk about storm-petrels you might interchange by seabirds (at least, in most cases).

**We have modified the introduction to widen the scope of the manuscript.**

Minor Comments:

6. Lines 50-51: You cite here Reed et al 1985 and Wilhelm et al 2021, but they don’t provide a list of species affected. In this sense, I feel more appropriate to state in this sentence that Procellariiformes is the most vulnerable with the number of affected species. In this sense, the most updated reference is the Chapter by Gilmour et al 2023 in the book Conservation of Marine Birds.

**L70-71 The statement and the reference has been updated to Gilmour et al 2023.**

7. Lines 71-72: I disagree with this sentence. There are at least another two studies where light reduction has reduced fallout and you have them included in the reference list: One is the pioneer study by Reed et al 1985 running a very similar experiment to yours, another is Rodríguez et al 2014 where fallout was reduced by turning the lights of a bridge off. I think that this information should be showed to readers in the introduction (as authors have very well done with the study by Miles et al. 2010).

**L80-81: We have removed this sentence and replaced it with “Efforts to reduce seabird strandings at common stranding sites by reducing ALAN have been successful with other storm-petrel and procellarid species. To our knowledge….” to highlight the valiant efforts by these authors.**

8. Lines 229-230: According to Table 1, fog is significant! Day of year may not be significant because the reasons given above.

**The paragraph above has been modified using the revised data results in Table 1 (see response to comment 9 below), to discuss all significant variables.**

9. Table 1. I do not understand why there are no estimates for all predictors nor why the dispersion model only includes fog. Are these the results of the GLMM?

**Table 1. We initially included the ANOVA table rather than the summary table. We have replaced the ANOVA table with the summary table which includes all the estimates. The best-fitting model included only ‘fog’ as the significant predictor of additional variance in the data. The statistics have now been updated.**

10. Line 280. It is the first time you call LEDs. It would be great to have a better description of the type of lights were on/off during the experiment. Critical aspects are intensity of the lamps and spectrum.

**We are attempting to obtain information on the intensity of the lamps and spectrum. We have now called the lights LEDs when discussing the lighting regime L108-109.**

11. Table 3. How do you assess the breeding status to complete the row ‘N of breeding individuals’? Should that information be at Methods?

**During the night surveys, breeding status was assessed by checking for the presence of a brood patch as outlined in Pollet et al. 2021. This was indicated in the methods L174.**

12. Lines 306-321: The think that the first paragraph is out of the scope of the ms: 1) I miss a discussion of the Reed et al 1985 study! 2) Lines 314-317, Telfer and co-workers study is not an experiment! 3) Lines 319: “…suggesting that they also exhibit positive phototaxis.” It seems like authors are re-inventing the wheel. I think that there is a great consensus on that seabird fledglings show phototaxis in flight. This has been demonstrated by GPS-tracking flights in Rodríguez et al 2015 Scientific Reports and 2022 Frontiers in Ecology and Evolution. In summary, I think that this first paragraph should be focussed on the reductions of fallout numbers by turning the lights off or shielding the lights described by Miles et al., Reed et al. and Rodríguez et al. (not sure if I am missing other critical studies).

**L330-344 We have updated the manuscript to discuss studies that have reduced ALAN as a form of conservation for grounded seabirds and we have shifted the focus of the paragraph as suggested.**

13. Lines 327-330: Although it might be useful for adults, please, note that the explanation about the reduced activity of fledglings (fledging) because high moon illumination is not a valid hypothesis, at least until new evidence arise. A full explanation is available in Rodríguez et al 2023 Conservation Science and Practice. So, please, reword to address the differences between age groups.

**L352-353 We have reworded the following sentence to differentiate between the adults and juveniles with regard to the two hypotheses.**

14. Lines 336-339: First, note that fog is significant according to table 1. Second, it seems that the second part of the sentence is contradictory to the first one.

**This sentence has been modified to reflect the updated statistical results.**

15. Lines 339-340: I would rewrite this part “ …most mass stranding events (63%)…” to something like “…six out of eight mass stranding events…” Confirm the numbers. Also about these lines and followings, could you give some information on the weather conditions? For example, was it rainy, cloudy or foggy during those six nights of mass stranding events?

**L361 The sentence has now been changed to say “six out of eight mass stranding events..” As for the weather conditions during these mass stranding events, I have inserted a table to show each mass stranding event and the weather conditions for each night.**

| **Date** | **# of birds stranded** | **Fog (yes/no)** | **Cloud cover (> 50%, < 50%)** | **Rain (yes/no)** | **Wind speed (km/hr)** | **Wind direction (onshore/offshore)** | **% moon illumination** |
| --- | --- | --- | --- | --- | --- | --- | --- |
| **Sep-30-2021** | **283** | **yes** | **NA** | **yes** | **39** | **onshore** | **37.0** |
| **Oct-01-2021** | **443** | **yes** | **NA** | **yes** | **NA** | **NA** | **28.0** |
| **Oct-02-2021** | **138** | **no** | **< 50%** | **no** | **42** | **onshore** | **19.0** |
| **Oct-03-2021** | **1100** | **no** | **> 50%** | **no** | **35** | **onshore** | **11.0** |
| **Oct-04-2021** | **108** | **no** | **> 50%** | **no** | **55** | **onshore** | **5.0** |
| **Oct-16-2022** | **188** | **no** | **> 50%** | **no** | **67** | **onshore** | **62.8** |
| **Oct-10-2022** | **167** | **yes** | **NA** | **no** | **44** | **onshore** | **33.8** |
| **Oct-19-2022** | **387** | **yes** | **NA** | **no** | **44** | **onshore** | **33.8** |

16. Lines 359: “…suggesting that…” What is the doubt? Since long time ago, it has been widely accepted that fledglings are more affected than adults.

**L384-385 We have changed the sentence to “...most birds found during this period were fledglings (Table 4), supporting the idea that they are the age class most susceptible to the effects of ALAN.”**

17. Lines 361: Be consistent with previous lines. 63% or 62.5%

**L387 Done.**

18. Lines 362-365: Sorry, I don’t follow the thread or your reasoning. Why are you proposing these two hypotheses? What is the evidence to propose the second hypothesis?

**The second hypothesis has been removed.**

19. Lines 372: Great!! “…based on brood patch presence.” This should be stated in Methods.

**This has been stated in the methods, L174.**

20. Lines 379: I would say that no eggs or nestlings would survive if one parent die. There should be plenty of references on petrels.

**L399-400 We have included a reference for Leach’s Storm-Petrels and updated this sentence to read “When a breeding adult dies, it disrupts a long-term monogamous pair bond** **(27) and the egg or chick will likely die…”**

21. Lines 399-401: “…while other results suggest that red and white light attract the most seabirds and migratory birds and cause the most disorientation in foggy conditions (43,46).” Please caution here! First, Syposz et al 2021 show that ADULT manx shearwaters are more deterred with white and blue lights (not attracted!). Adult is in capital letters to highlight the differences between adults and fledglings, which support my proposal of two models to each age group. Second, Poot et al. 2008 has been replied by Evans et al 2010 (https://ecologyandsociety.org/vol15/iss3/resp1) because of their experimental design and Poot and co-workers never replied. In addition, most evidence published after that paper indicate the opposite. So, I think that paper should not be cited anymore.

**L423-425 We have specified that Syposz et al 2021 refers only to adult manx shearwaters and have removed the citation for Poot et al 2008.**

22. Lines 408-409: Consider citing here Syposz et al 2021 as they found a smaller effect with shorter light treatments. To my best knowledge, it is the only paper testing that.

**L433 Done.**

23. Lines 413-415: Deploying an array of bird shelters deserve a paper explaining their potential use, pros and cons!! If you are not going to do it, I would ask for providing some information in this contribution on what is the proportion of birds found in the shelters vs the birds collected out, alive vs dead, etc. It seems as a potentially useful mitigation of light-induced mortality.

**While we agree that this would be an interesting paper, we are not able to determine the proportions collected in vs. outside of the bird shelters using our data as it was not recorded in the morning surveys where birds were rescued (inside or outside the bird shelters). Furthermore, when nightly surveys were conducted, the counts reported from the morning surveys immediately following are biased from the previous night's collection (and were therefore not conducted or removed from the models). A separate study would need to be run when birds were counted at night but not rescued, thereby allowing them to run into the bird shelters but also increasing their likelihood of being predated. Furthermore, we cannot monitor the whole perimeter of the plant simultaneously, which allows birds to be removed by predators or run into boxes before we can count them, or birds may land, then fly away, then land again in a different spot, thus increasing the likelihood of double counts.**

**Dear Taylor Brown,**

**Thank you for your helpful and thorough comments, we have taken care to integrate them into the manuscript. To help with the evaluation of our manuscript, we have included our responses to comments in bold and line numbers at the beginning of each statement from the revised document.**

Major comments:

1. Please note that there does not appear to be any public repository on Github under the username of “tori-burt”.

**Code/Software is now available at:** [**https://doi.org/10.6084/m9.figshare.25229471**](https://doi.org/10.6084/m9.figshare.25229471)

**Data files are now available at:** [**https://doi.org/10.6084/m9.figshare.25229414**](https://doi.org/10.6084/m9.figshare.25229414)

**Read me file is now available at: https://doi.org/10.6084/m9.figshare.25229528**

2. Light treatments are called “on” and “off” throughout the manuscript; however, because not all the lights were turned off at the seafood processing plant in the “off” treatment, I would suggest following the example of Miles et al. (2010) in calling the light treatments “on” and “reduced”. Although it seems like a minor change, someone skimming the paper might otherwise interpret the overall results as “strandings were reduced – but not eliminated – when lights were [completely] turned ‘off’”. Whereas, it’s more accurate to say that strandings were reduced when artificial lighting was reduced, which further suggests that perhaps strandings could have been eliminated if artificial lighting had been eliminated (so as to actually emphasize the importance of the manuscript’s results, rather than diminish it).

**Great suggestion. Thank you. We now refer to the “off” lighting condition as “reduced” throughout the manuscript.**

3. Additionally, it would be very helpful to me as a reviewer in assessing the study’s methodology at large and replicability in particular if a detailed (i.e., nightly) breakdown was provided of which treatment was applied (lights “on” or “reduced”) each night. E.g., this could be accomplished with a two-panel figure (one panel for each year), perhaps in the Supporting Information (including, ideally, the nights that nocturnal surveys were conducted). The authors mention there were some extended periods of the “reduced” light treatment (e.g., when the plant was shut down) which understandably could not have been avoided, but in its current form the manuscript does not give me a clear idea of the final lighting treatment schedule or of just how much of an effect these longer “reduced” lighting periods might have had on the results.

**We have now included S2 Fig. in the supporting information which outlines the periods of “on” and “reduced” lighting conditions at the plant throughout 2021 and 2022 and the number of strandings on each night.**

4. L30. This seems to imply that “positive phototaxis” is defined as or synonymous with “movement towards artificial light at night” when it could refer to movement toward any light at any time. I suggest minor rephrasing

**L28-29 We have rephrased the sentence to read “Nocturnal positive phototaxis, the movement towards artificial light at night (ALAN)...”**

5. L47-49. This sentence is good but there is a lot to unpack and it could benefit from a (brief) follow-up sentence or two explaining the perils related to stranding / rates of mortality following stranding. But this is very minor.

**L51-54 We have added details to emphasize the perils of stranding.**

6. L61. I might suggest also citing Imber (1975; Notornis) here (in addition to citation #15)

**L57 Done.**

7. L74-76. I just wanted to say that these are both extremely good points, and that is why it’s so great that the authors have done this current study!

**Thank you for the support of our study!**

8. L77. Spelling: “phototactic” vs. “phototaxic”

**L64 Done.**

9. L79-81. I might suggest a slight reformatting / rewording of this sentence to ensure it’s as clear as possible.

**L66-68** **The sentence now reads “In addition, more birds strand around sources of ALAN when cloud cover is high and moon illumination is low...”**

10. L106-107. I also mention this with regard to the figures, but I think it is worthwhile outlining the entire (or at least all of the major parts of) the lighting scheme of the whole building; not just the lights that were manipulated for the purposes of the experiment. I say this in part because from what I understand, petrels were picked up all around the building, not just on the NW side where the lights were manipulated (unless I am incorrect in my understanding of any of this). So the counts of birds stranded include presumably those affected (i.e., attracted) by the lights on the back of the building, and by the lights on the front of the building that remained “on” even in the “off” (reduced) treatment. So I think these un-manipulated lights are still relevant.

**We concur and though the vast majority of birds strand on the brightest wharf/harbour side of the plant, we have added a sentence that explains that location of other LEDs and affirm that birds also strand in other areas around and near the plant were checked in our nocturnal and morning surveys (L108-113).**

11. L123-24. Would it be possible to make the entire schedule (complete with each day’s actual rather than planned lighting treatment) available as Supporting Information, for the purposes of reviewing how balanced the schedule was?

**As mentioned above, S2 Fig. has been added to the supplemental information which outlines the lighting conditions at the plant across the year and how many storm-petrels were stranded on each of those nights.**

12. L124. For how long in total / how many 28-day periods in total (over the course of each year)?

**L127-129 There were approximately five 28-day periods in total as the schedule began on June 25 2021 and ended on November 10 2021. The start and end dates of the lighting schedule for 2021 and 2022 have now been added.**

13. L125-26. But did they, in fact, follow the schedule? Or did some nights deviate from the plan?

**L134 Some nights did deviate from the plan, and we have added a sentence to clarify this. Actual lighting conditions were always recorded when conducting both the nocturnal and morning surveys.**

14. L127-28. Similar to previous comments, this is where a schedule outlining the actual lighting treatment applied each night would be helpful as Supplementary Information. Otherwise it’s difficult to evaluate how well the lighting schedule actually allows the authors to test the hypotheses and whether there are any potential biases in the data as a result of lighting schedules (even if deviations from the schedule could not be helped)

**S2 Fig. Done.**

15. L128. How many lights are also on the back of the building? And how many lights were left on at the front of the building even during “dark” treatment nights? (As an aside, these various light locations could be shown in Figure 2 with a different symbol to represent the fact that they were always on)

**We have included the total number of lights on the building and the garage within the manuscript (L108-113) and in Figure 2.**

16. L129. Is it known what “briefly” constitutes here, on average or at maximum, roughly? Just out of curiosity.

**We do not have an approximate measure as it depends on the time required to unload the catch from the fishing vessels.**

17. L147. “the following evening” – to clarify: this is two evenings after stranding / capture?

**L152-154 This sentence has been revised for clarity.**

18. L149. Weather conditions on the morning of collection, or on the [previous] night of stranding? And including which variables exactly, and how were they measured?

**The fog, rain, and wind conditions from the previous night were recorded. These measurements were taken informally and based on observation of the weather at the time of recording. Weather conditions were not used in any formal analysis with the exception of fog (binary in the model), as it is difficult to get precise measurements of fog and its extent and duration.**

19. L149-50. Were birds collected from vessels? Or is this noted to determine when the lights were turned on? It might be useful to explain the significance of collecting vessel information at some point in the Introduction.

**While this is an important variable that likely influences strandings, we did not look at this in our study and it has been removed.**

20. L153-54. This point makes it especially of interest for me / readers to know the final lighting treatment schedule.

**Fig. S2 We included the figure you recommended.**

21. L157. What is the total estimated capacity of all the bird shelters combined? (this becomes useful information later, when considering predation that occurs before morning surveys / bird collection)

**While we can calculate the theoretical capacity of the shelters, the actual capacity of the shelters is difficult to gauge because it depends upon many factors. Birds may block the entrance or orient themselves to minimize space, and a bird going inside the shelter is contingent on the bird finding the shelter. Furthermore, bird shelters hardly ever reach maximum capacity and predation often happens within minutes of birds being stranded. Because of this, bird shelter capacity is not an accurate predictor of predation.**

22. L159-60. At the end of this line, it’s stated that there were 22 night surveys conducted in 2022; how many were conducted in 2021? Also, based on these lines it appears the timing of nocturnal surveys differed by year where in 2021 they were opportunistic and in 2022 they were approximately bi-weekly; but in the rest of the manuscript, these nocturnal surveys (from both years) appear to be collectively referred to as bi-weekly. I suggest omitting the term “bi-weekly” when collectively referring to the nocturnal surveys since it does not accurately describe the timing of nocturnal surveys for both years and since it could mean one of either “twice per week” or “twice per month” without the definition being provided.

**We have now included the sample size for 2021 and 2022 L165-169. We have changed the former “biweekly night survey” to “night survey” throughout the manuscript.**

23. L157-84. It would be pertinent to state somewhere in this section how many night surveys were conducted during lights “on” versus “reduced”, in both years. Because the method for deciding when to do nocturnal surveys differed between years, I would be concerned about one year having the surveys coincidentally falling on more lights “on” nights than “reduced” nights, and possibly affecting the results.

**L165-168 We have included the nights with lights on vs. off in both 2021 and 2022 for the night surveys. Across both years, the lights “reduced” condition is disproportionately represented and we address this in the manuscript on L175-176.**

24. L164. What was it about the condition of the feathers that indicated they were juveniles?

**L172-173 The tips of the juvenile primary feathers are more pointed, less worn, and blacker than adult primary feathers. The sentence has now been changed to “darker colour and non-weathered condition of the primary feathers indicates juvenile birds..”**

25. L171-72. Please clarify if possible, as I’m not sure I understand the meaning here, in the context of the rest of the sentence.

**L176-178 We have clarified this section.**

26. L177-78. Same as in the daily survey methods section, I would like to know the relevance of including the vessel data.

**We agree that vessel data are not relevant to the current analyses and they have been removed from the manuscript.**

27. L179. Repeats from lines 159-60; could be combined / condensed

**L159 has been condensed with L179.**

28. L182. Is it not known exactly how long the lights were off for this time period? If not known, how were daily survey stranding numbers matched to the treatment used?

**When daily morning surveys were performed, the lighting condition at the plant was recorded. We are not able to isolate the exact period because the light schedule was not always followed, therefore, we cannot pinpoint the exact period when the plant was shut down.**

29. L189. Please clarify if this includes birds found both alive and dead.

**L188-189 We have clarified this throughout the manuscript.**

30. L191-92. Is this an established method used in (an)other study/ies for which a citation can be provided? Or how was this method decided upon, to determine the peak stranding period?

**We chose this threshold based on manual examination of the data. We chose the mean specifically because the median was zero.**

31. L193-211. Please clarify that in this analysis, only daily / morning survey data were used.

**L194 Done.**

32. L201-02. The data used were from which location (St. John’s?) and at which time point? Also, this sentence says “weather information” is from Custom Weather Inc, but once wind speed and direction are removed (which were apparently obtained from the government of Canada), the only weather variable left in the above list of variables is fog; was this the only variable gleaned from Custom Weather Inc? If so, the fog data originate from what location? I am partly curious because fog can be extremely localized and I would be very cautious about taking fog data from St. John’s to apply to Bay de Verde.

**The weather data from St. John’s were used to estimate cloud cover for the night surveys. Weather information from Custom Weather Inc were not used in the daily morning surveys to estimate fog. Mention of Custom Weather Inc has been removed from the daily morning survey paragraph.**

33. L203-04. Wind data are for St. John’s, or elsewhere? And wind data were used from which time point(s) – a certain time of the night during which strandings occurred (i.e., the night before bird collection)?

**L206-208 Weather data were recorded from a weather station in Grates Cove, NL (13 km from Bay de Verde, NL) and wind speed was the max gust recorded per day. Wind direction was the direction of that max gust recorded. We have changed the previous sentence to now read “Wind speed (max gust per day) and direction information from a weather station in Grates Cove, NL (48.1675, -52.9393; 13 km from Bay de Verde, NL) were obtained from the Government of Canada…”**

34. L201-06. So, just to be clear, none of the weather data collected on the mornings of bird collection were used in the analyses (see line 149)? If this is the case, I might suggest omitting mention of collecting these data at all (and omit mention of any other data collected that were not used, if applicable).

**Mention of the collection of irrelevant weather information has been removed.**

35. L209-10. Were these events (>100 birds) considered statistical outliers? Or how was “outlier” defined? How many of these events were there over the course of the study, just to give some context?

**These events were considered statistical outliers, we chose the threshold of >100 birds based on the distribution of the data. There was a very large gap in observations of nights where the number of stranded birds was in the 10s and nights where the number of stranded birds was an order of magnitude higher.**

36. L212. Please clarify if this analysis includes birds found both alive and dead.

**L198-199 Done.**

37. L214. It is stated in line 178 that fog data were collected during these surveys; please double-check.

**This has been clarified, see answer above.**

38. L215. What is considered “supplementary data”, and how were they used to estimate cloud conditions? Or were the St. John’s cloud data simply used as-is?

**Weather data received from Custom Weather Inc. were broken into multiple weather descriptors (ex. broken clouds, mostly cloudy, clear). We labelled any descriptor that indicated cloud cover was medium - high as greater than 50% cloud cover and any descriptor that indicated cloud cover was low was labelled as less than 50% cloud cover.**

39. L218. See comment re: “outliers”, lines 209-10.

**This has been addressed above.**

40. Results. The p-value column(s) in the two tables contain a mixture of exact p-values and significance levels, and the way they are reported in tables does not always match the way they are reported in-text. Please double-check consistency and ideally state somewhere which significance level you used as a cutoff.

**L235 The significance level has been clarified.**

41. L223. I believe the chi-square value of 6.73 might actually round to 6.74 based on Table 1. Also I suggest references to the appropriate tables be made in the brackets with test statistics, where applicable.

**Done.**

42. L224. Is it a 95% confidence interval that’s being presented? If so, I would suggest adding “95%” before the “CI”, in the brackets (and throughout, where applicable; e.g., L246).

**This has been revised throughout the manuscript.**

43. L228-29. Since this sentence is about “birds stranded per night” being higher in 2021 than in 2022, might it be more appropriate, instead of reporting total strandings per year, to report the average number of birds per night?

**L240-241 We have revised the sentence to include the average number of birds per night.**

44. L229-30. Fog – from Table 1, it does appear that fog is significantly associated with the number of stranded birds, at least to p < 0.001, unless I am misunderstanding the results. Please double-check.

**The paragraph has been modified to discuss all significant variables.**

45. L231. I would suggest perhaps including the test statistic(s) and p-value here as well as in the table. (See also L251)

**We have included references to the table in the text with the appropriate statistic.**

46. Table 1. If applicable, I suggest clarifying in the caption that this table presents results from the daily / morning surveys only

**Table 1. Done.**

47. L245. How are the “later months of the breeding season” defined for the analysis? I suggest detailing this in Methods > Statistical Analysis, unless it actually refers to “day of year”, in which case I would suggest changing the wording here to specifically reflect that.

**The wording has been changed to reflect the use of day of year throughout the manuscript.**

48. Table 2. Similar to Table 1, I suggest clarifying in the caption that this table presents results from the nocturnal surveys only

**Table 2. Done.**

49. L262-63. Similar to comment on L245, I might suggest using slightly more precise wording so as to avoid confusion. I assume “seasonal effect” refers to the effect of “day of year” on stranding numbers, since there was no variable based on “season” used in the models.

**This has been revised throughout the manuscript.**

50. L266-67. “Body part estimations” have not been introduced or explained in the main text of the manuscript thus far. If the authors want to keep it in the main text, I would suggest bringing the methodology over from the Supporting Information to the main text. However, I would actually instead suggest omitting it from the main text. In this case, including body part estimations (which were only conducted four times during the whole study and half of those times during the peak stranding period) in calculating the percentage of birds stranded during the “peak stranding period” seems to me like it would only introduce unnecessary noise into the calculation.

**We included these measurements to show that the presented counts are gross underestimations, to improve clarity the body part estimation has been removed from the main text and is instead included in the supplemental material.**

51. L271. Does this “error” refer specifically to Standard Error?

**L293 It refers to a 95% CI, the wording in the caption has been changed from “error” to “95% CI.”**

52. L272. “outliers” – see comment L209-10.

**These values (387 and 283) were considered statistical outliers.**

53. L276. Is “body part count” the same as body part estimation? How defined? See comment on L266-67.

**The body part count is the same as the body part estimation. As mentioned above, it has now been removed from the main text and is instead included in the supplementary material.**

54. L278-79. Similar to comment on L266-67, I feel that including body part counts in this calculation of % alive only adds unnecessary noise, since the body part counts were not conducted during every survey.

**Addressed in the comments above.**

55. L279-80. When I add 1950 (birds stranded when lights on) and 1929 (birds stranded when lights reduced), I get 3879 birds total. Even if I add to this the 300 birds estimated from the body part counts, I only come to a total of 4179 birds. I’m not sure if I am doing the math incorrectly, but this does not equal the total given in L277 of 4225 birds; perhaps a bit of clarification would be helpful.

**During the analyses, some of the observations had to be excluded due to data unavailability for one or more variables (ex. no lunar data). However, in the final counts of stranded storm-petrels, all observations were included. The number of observations removed to proceed with each analysis have been included in the methods section (L158-159, L168-169, L199-202, and L227-229) and the sentence including the final count has been revised to note that it includes previously excluded observations.**

56. L282-84; Table 3. I would suggest specifying here exactly how many birds were “examined” in total (including, presumably, not only adults and juveniles but also unknown-age birds). As written, it appears that a total of 1859 birds were aged (1609 juveniles + 250 adults), and all of them were assignable to either juvenile or adult (because 86.6% + 13.4% = 100%). But in that case it becomes unclear where the number 731 comes from for “unidentified” (unknown age) birds in Table 3. I also suggest changing “examine” (L283) to “assign age to” (or something similar), for clarity.

**L306 We have replaced “examine” with “assign age to.”**

57. One other thought / question I have is, could the bias in 2021 toward conducting nocturnal surveys during the peak stranding season (when it is known they are mostly juveniles) cause a bias toward proportionally more juveniles than if nocturnal surveys had been conducted evenly throughout both 2022 and 2021?

**L306-307 Good point. We have updated this estimate to just include 2022.**

58. L284. What percentage of adults stranded from June to August?

**L305 64.6% of adults stranded during this period. This has been added.**

59. L288-89. How are these time periods defined exactly (“fall” and “breeding season”) for the purposes of these results? Does this mean the nocturnal surveys from June 2021 are omitted (and if so, why)? If “fall” and “breeding season” are not strictly relevant, I would suggest omitting and just saying 2021 and 2022.

**Table 4. Reworded the table caption for clarity.**

60. Tables 3 and 4; also throughout. For clarity, I suggest changing “unidentified” to “unknown-age”.

**This has been revised throughout the manuscript.**

61. L300-01. In referring to such a small number of nights of mass strandings (8 total), instead of using % (e.g., 62.5%, 75%) I would suggest using raw / whole numbers.

**This has been revised throughout the manuscript.**

62. L302-03. Is it meant here that 6/8 mass stranding events occurred on nights of nocturnal surveys, and 2/8 occurred based on the more limited evidence of morning surveys? I’m curious what the authors think about the fact that morning surveys are a different methodology that misses a lot of the birds that come in, therefore probably “missing” some nights that would otherwise be considered “mass stranding events” (>100 birds).

**The morning surveys would miss mass stranding events which occurred at night which is why we supplemented the data with the nocturnal surveys; this is detailed on L163-164.**

63. L316-17. It is unclear to me what the main take-home message is of this sentence without knowing how this statistic relates or compares to the other years of that study (i.e., without additional context). Were 1982 and 1983 exceptional somehow in the proportion of shearwaters found in brightly illuminated locations? Or was the proportion high in all years? Are the years relevant, or could an overall proportion be provided for the entire study?

**This paragraph has been reworded.**

64. L317-19. That any number of birds was grounded anywhere does not suggest positive phototaxis in itself; I suggest the authors add some sort of direct context related to light that would support the suggestion of positive phototaxis more directly.

**The suggestion of positive phototaxis has been removed from this sentence.**

65. L319-20. I suggest omitting or altering the first clause of this sentence (before the semicolon) because the meaning is somewhat unclear. I initially interpreted it as either the population increasing as the result of reduced ALAN, or that they responded with attraction (“positive” phototactic behaviour) toward reduced ALAN; only after reading the rest of the sentence do I realize that neither of those meanings was intended by the authors.

**This sentence has been revised to remove the word “positively” to improve clarity.**

66. L322-23. Please consider adding a couple words to specify that this sentence refers to the current study.

**L329 Done.**

67. L346. I understand from the Methods and Results that cloud cover data were indeed obtained and used in analyses. Please double-check and/or clarify this sentence based on that.

**This has been clarified above.**

68. L336-39. I think this sentence could benefit from rephrasing for better flow and clarity.

**This sentence has been revised for clarity.**

69. L341-44. This is very interesting! I would genuinely be curious as to what the authors suggest as a good next step in the research on this topic. Do the authors have a hypothesis underlying this point about the relationship between cloud cover / fog and mass strandings?

**We are recommending long-term studies to understand episodic mass mortality events rather than research into fog and cloud cover. Fog and cloud cover influences are highly variable depending on the density of the fog, light source and direction, and avian vision. Investigation into this concept is beyond the scope of what we are proposing.**

70. L345-70. The train of thought in this (quite large) paragraph seems to me to be somewhat murky in the sense that I’m not sure what the main take-home message(s) is/are, and the main topic seems to shift throughout. I suggest breaking into 2+ separate paragraphs, each with its own take-home message; e.g., one on mortality, survival, predation levels, and how nocturnal surveys capture a different picture of stranding numbers than morning surveys; and another on fledglings versus adults being differentially affected by light and why, taking into particular consideration the fact that mass stranding events occur just as often (or more often) in reduced lighting conditions.

**We have now partitioned the paragraph into two paragraphs; one addressing the differences between the survey types and the other discussing the peak stranding period and the age effect.**

71. L345. I’m not sure that including “breeding season” here is necessary, as it might just add confusion for the reader. The entire time storm-petrels are present in the area is the breeding season.

**The phrase “breeding season” has been removed.**

72. L347-50. I suggest briefly describing the implication of this finding.

**Thank you for your comment. We feel that we have adequately addressed this point in the following two sentences.**

73. L345-54. These lines could probably be condensed and made more concise, and some additional discussion of the body part counts could be both interesting and relevant here (if desired).

**Done.**

74. L356. “season” – see comment on L245.

**Removed the word “season.”**

75. L356-57. Please specify whether this is generally true across latitudes / breeding locations, or if it’s specific to Newfoundland.

**L383 Included that this is specific to Atlantic Canada.**

76. L360. Please clarify if “these stranding events” refers specifically to the 8 documented mass stranding events or to strandings in general.

**L386 Done.**

77. L363-64. The current study already shows that a partial reduction in lighting is enough to reduce strandings; I wonder if “eliminate” would be more accurate than “reduce”.

**L390 The word “reduce” has been replaced with “eliminate.”**

78. L364-65. I’m not sure that I understand the rationale for the second hypothesis. It would be helpful if the authors could clarify how the results outlined in L360-61 support this hypothesis (as it is said in L362-63 that the results could be interpreted as support for it; but in L365-66 it is said that results concur with the first hypothesis).

**The second hypothesis has been removed and the sentence has been rephrased for clarity.**

79. L365-67. These lines appear to repeat what was said in L354-60. Please double-check.

**L365-67 have been removed.**

80. L367-69. These lines appear to partially repeat what was said in L340-41; perhaps they can be combined / condensed.

**The sentence (L367 - 369) has been removed.**

81. L369-70. If I understand their intended meanings correctly, perhaps the forward slash and comma can be replaced with “÷” and “=”, respectively.

**See comment above.**

82. L373-74. This sentence could be taken to mean that the number of stranded breeding birds in general may have been underestimated, but I doubt that was the meaning intended by the authors since counts of found stranded birds are almost by definition underestimates. I suggest the wording be altered slightly to clarify the intended meaning to be about the proportion of stranded adult birds that were verified as breeders.

**L379 This sentence has been reworded for clarification.**

83. L375. It might be helpful here to tell the reader when hatching typically occurs in this species in Newfoundland

**L395 Done.**

84. L371-83. It might be worth mentioning / reiterating in this paragraph the population declines at Baccalieu and/or the threatened status of the species, therefore reinforcing the importance of reducing adult mortality related to positive phototaxis

**L404-406 Done.**

85. L385-89. Very cool study. It sounds to me like social media reports of stranded birds throughout the province could help researchers find those stranding-prone locations and times to target for conservation efforts. A more explicit connection between those two concepts could be made here.

**Thank you!**

86. L389-91. “Conservation efforts at the seafood processing plant… are effective elsewhere” implies that the efforts at the seafood processing plant are somehow affecting strandings and mortality at a different site. Please consider re-wording slightly.

**L412-416 Done.**

87. L399. Please specify whether this finding was related to passerines only, and clarify if the attractive effects of blue and green light were not observed in clear conditions.

**L421-423 Done.**

88. L397-402. Just a note here to clarify that different light sources are really just different spectra of light, the same way that blue, green, red, and white light (L398-400) are different spectra of light that can vary even within a “colour” of light; they are all just collections of wavelengths expressed at different intensities. So I’m not sure that distinguishing between “wavelength” and “light source” as two different variables that may affect attraction and stranding is fully accurate.

**L420 We have added the word “peak” to provide clarity to the reader. When we refer to differences in light source, we mean at the same wavelength of peak relative intensity, there may be differences in the emittance of other wavelengths across the light spectrum (LED vs. metal halide). When we mention “wavelength”, we mean differences in where the peak emittance occurs (500nm vs 700nm, colour).**

89. L405. “LESP” as an acronym has not been defined in the manuscript; please use full name or define the acronym.

**Done.**

90. L415-16. The Puffin and Petrel Patrol (PPP) itself is only a program, but it is run by a non-profit organization (CPAWS-NL).

**L439 Reworded for clarity.**

91. Figure 1. Are both map panels oriented North-up? A North indicator would be useful.

**Figure 1. Done.**

92. Figure 2. See also comment on L106-07. A separate symbol to indicate the locations of un-manipulated lights (e.g., on the back of the building and along the wharf) would be helpful in allowing the reader to see by how much the lighting was reduced in the reduced (“off”) light treatment. Since bird shelters are located along the back of the building and birds were presumably also collected there, it seems pertinent to include this information (in part also for replicability).

**We have now included all lights around the plant. It is specified in the figure caption (Fig 2) and in the text (L108-113) that only the lights at the front of the plant are being manipulated.**

Supporting Information

93. SI. L16. “to approximate a measure of mortality when researchers were not present” – please clarify that the intended meaning is to quantify mortality that occurred between each date of body part counts, and how accurate these counts might be given the possibility of body parts disappearing in the interim.

**L625 We have clarified the intended meaning. We cannot comment as to how accurate the counts are to the actual mortality at the plant as we do not know how many body parts are lost due to weather conditions or taken by predators. As we emphasize throughout the manuscript, these counts are reporting minimum numbers of stranded birds and simply meant to highlight the level of mortality occurring at the plant.**

94. SI. L26-28. A brief description of what (generally speaking) is being calculated would be helpful to include before defining the “days” variable and the formula.

**L633 Done.**

95. SI. L29. I suggest using a more precise term than “lost”.

**Done.**

96. SI. L23-32. Please define what a “collection period” is and, in L31, specify the number of collection periods used in the calculation. Also, were the body parts collected on September 13 excluded from the final calculations of estimated mortalities that occurred between September 13 and October 14? Some additional clarification of the calculations described in L29-32 would be helpful.

**L641-644 We have defined the collection period and the number of collection periods used in the calculations. The body parts collected on September 13 were excluded from the final calculations and this has now been included in the text (L42-43).**

97. SI. L34-35. Would the authors please consider including the numbers of each type of body part found on each collection date, so that the reader can confirm the results make sense given the calculations described in SI L23-32.

**L642-644 Done.**
