# Supplementary material for: Reduction of coastal lighting decreases seabird strandings
Source: PLoS One. 2024 Jun 5;19(6):e0295098. doi: 10.1371/journal.pone.0295098 (PMC11152301; doi:10.1371/journal.pone.0295098)
Supplement: Attachment — Submitted filename: Response to Reviewers.docx [file pone.0295098.s009.docx]

Response to Reviewers - PONE-D-23-37663

**Dear Taylor Brown,**

**Thank you for your thoughtful comments on the revised manuscript. To help with the evaluation of our manuscript, we have included our responses to comments in bold and line numbers at the beginning of each statement from the revised document.**

Minor comments:

1. L108-13. Here and in Figure 2, it still appears that some of the lights along the northwest side (the “front”) of the building that were un-manipulated are not mentioned or included in the overall count of how many lights are present in the immediate area of the building. Specifically, in the “reduced” lighting photo (Figure 3b) I can see five pairs of LED lights (so ten total) along the wharf, plus a cluster of four bright LED lights on the far right side of the building and two apparent High Pressure Sodium pole lights in the middle of the picture beside the wharf.

**L108 – L115 There are 16 overhanging LED light along the wharf-side of the plant. All were turned off except for four at the far end of the plant when boats unloaded their catches there. The high-pressure sodium lights and the wharf lights were always turned on. We have re-counted the lights at the plant, updated the diagram, and have changed the reported lighting count in the manuscript.**

2. L241-42. Fog was also not significantly associated with the number of stranded birds and could be added to this sentence.

**L244 – L245 Done.**

3. L254-62. Thank you for specifying adults in L254. It may help the reader if the rest of the paragraph also refers specifically to “stranded adults” or “stranded adult birds” rather than the more general “stranded birds” or “stranded storm-petrels”. Similarly, in L272 juveniles are specified but the rest of the paragraph could also refer specifically to juveniles for utmost clarity (L272-77).

**This has been updated throughout the manuscript.**

4. Table 4. Please clarify in the table caption if means are accompanied by standard deviation.

**Table 4. Done.**

5. L346-48. I suggest clarifying that “fewer storm-petrels of [all age classes] stranded when moon illumination was high…” and either remove or change the wording of the second clause which refers to juveniles specifically, because of the non-significance of that result. The morning survey data (indicating a significant effect of moon illumination) have much higher resolution than the night survey data anyway, so greater emphasis / confidence could be placed on that result.

**L349 – L352 Good point! We do want to note that there was a trend in juvenile birds, but we do not want to mislead readers. We have added a section clarifying that results from the morning surveys were significant and results from the night surveys were not significant for both adult and juvenile models before highlighting the trend in juvenile birds.**

6. L352-55. The grammatical structure of this sentence could be improved slightly for readability.

**L357 – L359 Done.**

7. L357. Perhaps start a new paragraph here for wind and other weather effects.

**L363 Done.**

8. L386-90. Is it possible to somehow combine this section with L365-69, or condense it, since both sections cover the potential causes of mass strandings and how they relate to light condition? This could be achieved, in part, by shifting the intervening paragraph (L370-78) down so that it immediately precedes the paragraph that currently starts at L391 (about the importance of adult mortality). This flow of ideas would seem to me to be more logical. Also, L388-89 appears to imply that a potential explanation for the random nature of mass stranding events (with respect to lighting condition) is due to a stronger phototactic response by fledglings; but if it is random, then I would assume there is no light-related explanation.

**We have condensed the sections together on L388 – L394. The reason we proposed this explanation is because it has been well-documented that fledglings demonstrate stronger positive phototaxis relative to adults. Therefore, they may be attracted to unmanipulated lights that were turned on at the plant, regardless of the lighting condition in our experiment, (wharf lights, shed lights, etc.). We have clarified the meaning of our explanation on L391 – L394 and removed the word “random” for clarity.**

9. L442. This is the first time that “puffin” appears in the manuscript, so I suggest including the binomial species name.

**L446 Done.**

10. Figure S2A. There appear to be two triangular (night survey) points in October that lack colour to indicate lighting condition. Is this a mistake, or is light condition unknown? Overall, I very much appreciate the addition of this figure to the supporting information! It’s nicely done.

**Figure S2A The lighting condition was unknown, and those points should not have been included. They have been removed from the figure.**
